# P-680. Etiologic investigation by targeted next-generation sequencing for community-acquired pneumonia of unknown etiology in children

**DOI:** 10.1093/ofid/ofaf695.893

**Published:** 2026-01-11

**Authors:** Ki Wook Yun, Dayun Kang, Seung Ha Song, Ye Kyung Kim

**Affiliations:** Seoul National University Children's Hospital, Seoul, Seoul-t'ukpyolsi, Republic of Korea; Department of Pediatrics, Seoul National University Children’s Hospital, Seoul, Seoul-t'ukpyolsi, Republic of Korea; Department of Pediatrics, SMG-SNU Boramae Medical Center, Seoul, Seoul-t'ukpyolsi, Republic of Korea; Seoul National University Children's Hospital, Seoul, Seoul-t'ukpyolsi, Republic of Korea

## Abstract

**Background:**

Despite advancements in diagnostics, many pediatric community-acquired pneumonia (CAP) cases lack identified pathogens. We aimed to characterize CAP of unknown etiology (CAP-UKN) in children by applying broad-panel targeted next-generation sequencing (tNGS).

**Fig. 1.**
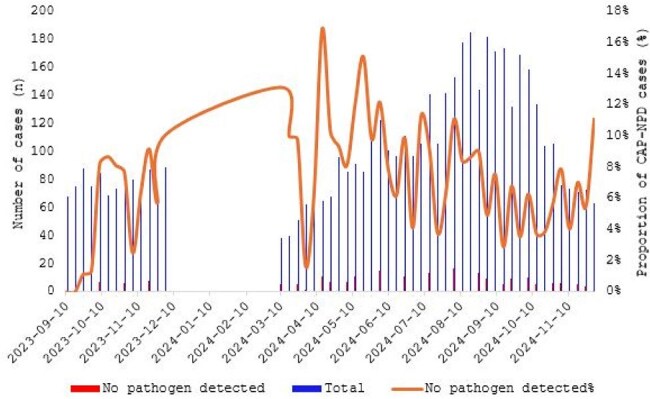
Epidemiology of CAP with no pathogens detected in Korean children based on weekly discharge monitoring

**Fig. 2.**
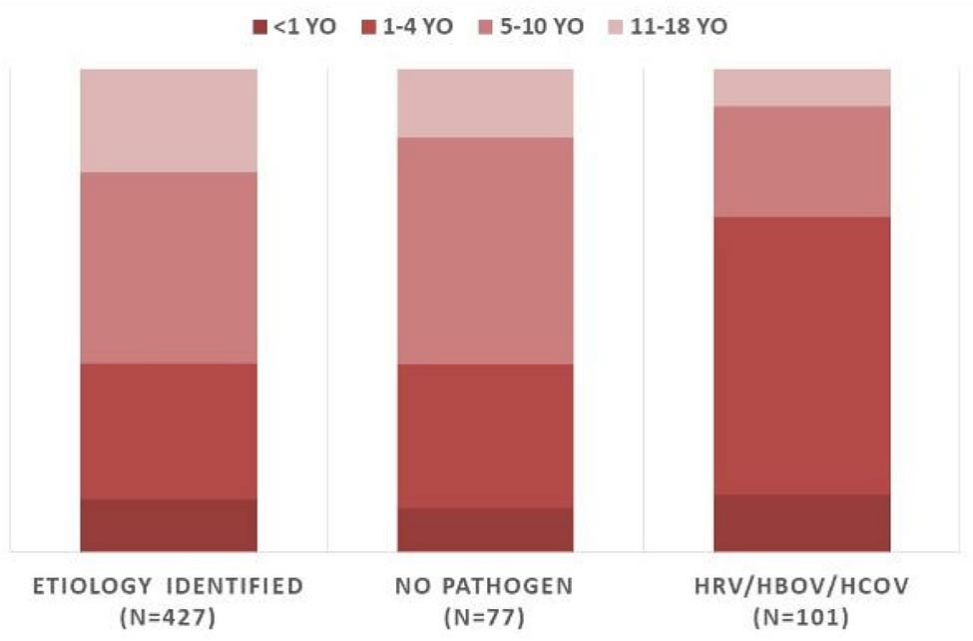
Age group distribution and CAP pathogen detection in children

**Methods:**

From September 2023 to November 2024, a prospective surveillance study was conducted across 26 hospitals in Korea. CAP-UKN was defined as cases with no detected pathogens or detection of only human rhinovirus (HRV), human bocavirus (HBoV), human coronavirus (HCoV), or common colonizers. Residual respiratory specimens were analyzed using 16S rRNA/internal transcribed spacer (ITS) sequencing and tNGS.

**Fig. 3.**
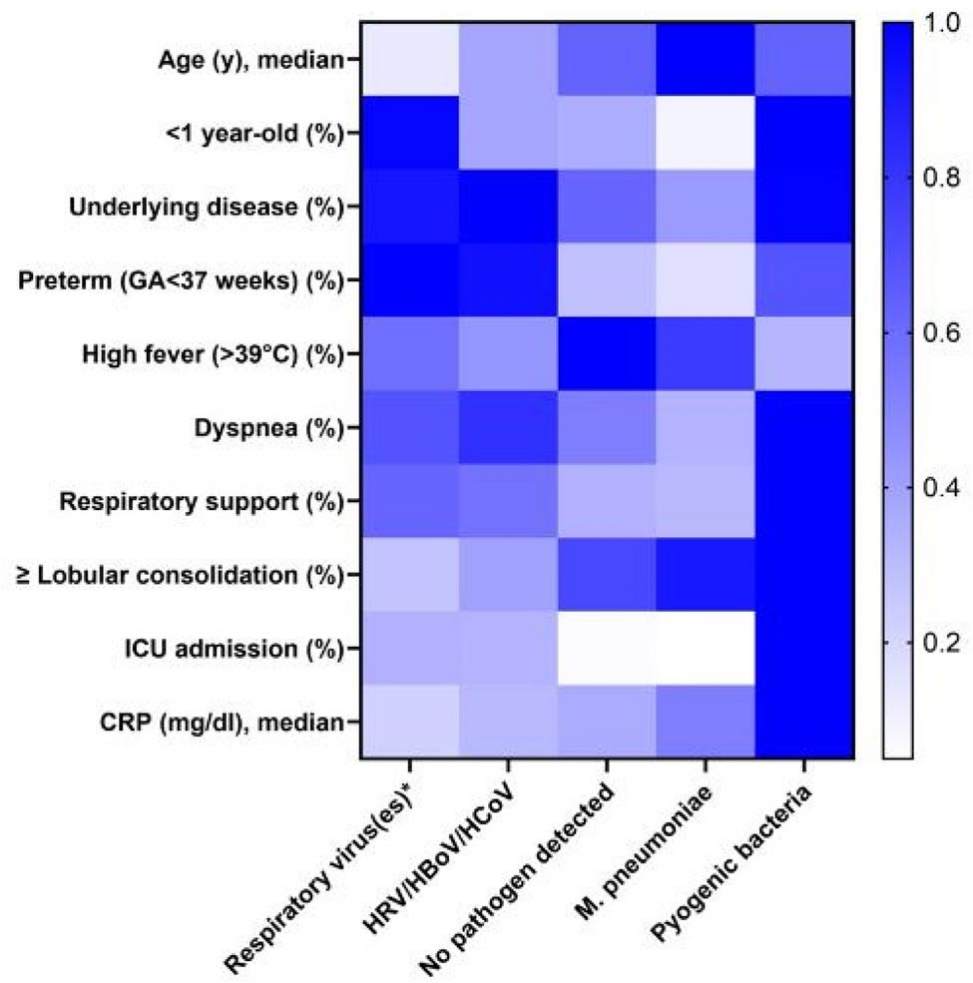
Comparative heat map of clinical characteristics based on etiologic pathogens identified

**Table 1.**
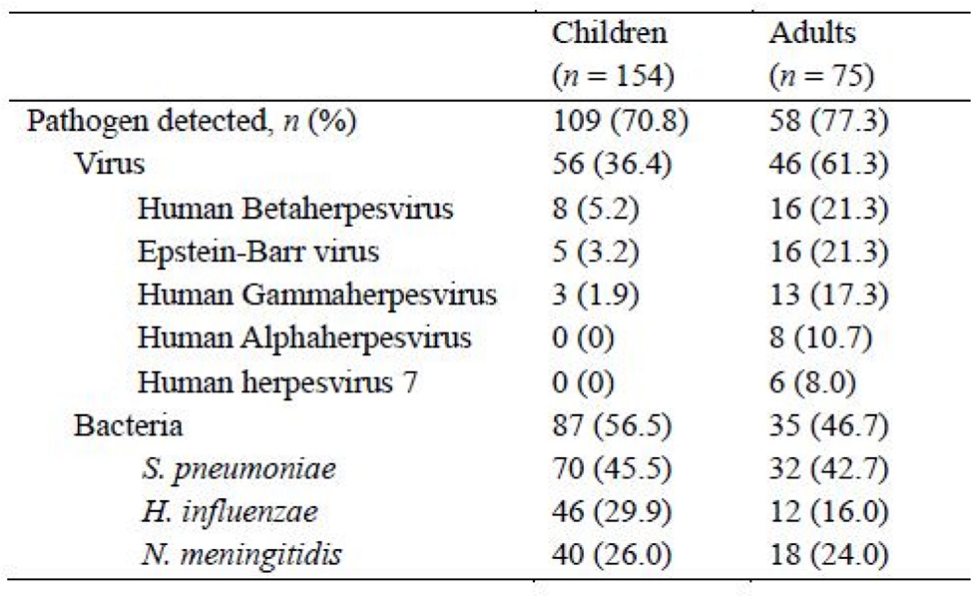
Viral and bacterial pathogens detected by mNGS in patients with CAP of unknown etiology

**Results:**

Among 605 pediatric CAP cases, 178 (29.4%) were classified as CAP-UKN, including 77 (12.7%) with no pathogen detected (CAP-NPD). CAP-NPD was more common in children aged 5–10 years (46.8%) and showed clinical features similar to *Mycoplasma pneumoniae* pneumonia, whereas HRV/HBoV/HCoV-positive cases were more frequent in children aged 1–4 years (57.4%) and resembled viral pneumonia. tNGS identified additional pathogens in 70.8% of CAP-UKN specimens (109/154 in children). *Streptococcus pneumoniae* was detected in 45.5%, *Haemophilus influenzae* in 29.9%, and *Neisseria meningitidis* in 26.0% of pediatric cases. Human betaherpesvirus was the most common viral pathogen (5.2%), followed by cytomegalovirus (4.5%) and SARS-CoV-2 (3.9%). By 16S/ITS sequencing, additional bacteria and/or fungi were detected in 23.8% of children and 56.7% of adults. Most were commensal flora such as *H. influenzae, Moraxella catarrhalis*, and Viridans streptococci. Compared to pediatric patients, adults showed higher detection rates of pyogenic bacteria (36.0% vs. 11.7%) and herpesviruses (61.3% vs. 36.4%) and experienced more severe outcomes, including ICU admissions (29.3% vs. 5.3%) and invasive ventilation (22.6% vs. 4.0%).

**Conclusion:**

Pediatric CAP-UKN may involve undetected atypical pathogens or overlooked respiratory viruses, including herpesviruses. Broad-panel tNGS enhances pathogen detection, offering important insights into the complex and previously unrecognized etiologies of pediatric CAP.

**Disclosures:**

All Authors: No reported disclosures

